# A Novel Membrane-like 2D A’-MoS_2_ as Anode for Lithium- and Sodium-Ion Batteries

**DOI:** 10.3390/membranes12111156

**Published:** 2022-11-16

**Authors:** Ekaterina V. Sukhanova, Liudmila A. Bereznikova, Anton M. Manakhov, Hassan S. Al Qahtani, Zakhar I. Popov

**Affiliations:** 1 Laboratory of Acoustic Microscopy, Emanuel Institute of Biochemical Physics RAS, 119334 Moscow, Russia; 2 Aramco Innovations LLC, Aramco Research Center, 119234 Moscow, Russia; 3 EXPEC Advanced Research Centre, Saudi Aramco, Dhahran 31311, Saudi Arabia; 4 Academic Department of Innovational Materials and Technologies Chemistry, Plekhanov Russian University of Economics, 117997 Moscow, Russia

**Keywords:** DFT, VASP, TMD, LIB, SIB, MoS_2_ membrane

## Abstract

Currently, new nanomaterials for high-capacity lithium-ion batteries (LIBs) and sodium- ion batteries (SIBs) are urgently needed. Materials combining porous structure (such as representatives of metal–organic frameworks) and the ability to operate both with lithium and sodium (such as transition-metal dichalcogenides) are of particular interest. Our work reports the computational modelling of a new A’-MoS_2_ structure and its application in LIBs and SIBs. The A’-MoS_2_ monolayer was dynamically stable and exhibited semiconducting properties with an indirect band gap of 0.74 eV. A large surface area, together with the presence of pores resulted in a high capacity of the A’-MoS_2_ equal to ~391 mAg^−1^ at maximum filling for both Li and Na atoms. High adsorption energies and small values of diffusion barriers indicate that the A’-MoS_2_ is promising in the application of anode material in LIBs and SIBs.

## 1. Introduction

Metal-ion batteries are currently the main power source for electronic devices due to their high specific capacity [1,2]. Lithium-ion batteries (LIBs) are the most widely used [3] for many reasons such as their high energy density and the absence of memory effects [4]. Meanwhile, the widespread availability and low cost of sodium, which has a close value of redox potential (only ≈0.3 V higher than lithium) and is characterized by the similar chemistry of ion intercalation, make sodium-ion batteries (SIBs) no less attractive than LIBs [5,6]. On the one hand, SIBs can serve as an excellent alternative to the LIBs taking into account the reduced availability of lithium and some shortages affecting their application [3,7,8]. On the other hand, Na^+^ ions have a larger radius, which directly affects the mass transfer and the energy storage in the electrochemical process [9].

Layered bulk structures consisting of two-dimensional monolayers can act as effective anode materials due to the rapid transport of ions in the interlayer space and the large surface area which improves the adsorption of particles [10,11,12,13]. For example, at this moment, graphite, with a layered structure, is the most widely implemented anode material for LIBs [14] and is characterized by a high theoretical capacity of 372 mA·h·g^−1^ for Li atoms (by forming LiC_6_) [15]. Graphite electrodes have a long service life [16]; however, they are characterized by a low range of operating temperatures [17]. In the case of SIBs, the graphite’s value of electrochemical capacity is less than 35 mA·h·g^−1^ [18,19] which was attributed to the fact that the graphite’s interlayer distance (~0.34 nm) is too small to accommodate Na^+^ ions (the minimum required distance is 0.37 nm [20]). Moreover, the Na–C chemical bond is less covalent compared to the Li–C bond [21], and, as a result, Na–C compounds are thermodynamically unstable [21,22]. However, alkali-metal ions with large atomic numbers (such as K^+^ ions) can form energetically favorable configurations in graphite sheets [23,24], but the high values of these ion masses make their use in metal-ion batteries less favorable. In this regard, current research is focused on the development of new electrode materials, first, to overcome limitations and eliminate, for example, shortcomings in both LIBs and SIBs [25] and second, to improve the characteristics of the existing devices. Carbon-based materials are an attractive platform to enhance metal-ion battery performance. For example, yolk–shell ZnS@C structures for Li- and K-ion storage [26] and porous FeS@N-doped carbon nanosheets for Na- and K-ion storage [27] demonstrate high capacity, but their use requires complex approaches to the synthesis.

The transition-metal dichalcogenides (TMDs) are applicable for both LIBs and SIBs [28]. The interlayer space in bulk TMDs and the weak van der Waals forces between the layers provide not only a large number of intercalation channels for metal ions but also a rapid diffusion of particles between the layers [29,30,31]. However, TMDs are characterized by lower theoretical capacity compared with graphite due to the larger molecular weight of the elements forming the structure. For example, bulk 2H-MoS_2_ has a value of 167 mAh·g^−1^ (considering one Li atom per one MoS_2_ formal unit) [32]. This capacity can be increased by two times by switching to a monolayer MoS_2_ (two Li atoms per one MoS_2_ formal unit) [33]. However, a further increase of capacity characteristics requires the application of artificial approaches, such as composite construction [34,35]. The second possible approach is the complete decomposition of MoS_2_ accompanied by the formation of Li_2_S (Na_2_S) and Li-Mo (Na–Mo) alloys. However, this method will complicate the reverse transition and reflect poorly on the cyclicity [32,36,37]. In addition, there is an alternative approach which is the search for new phases of TMDs having less density than H-MoS_2_ monolayers because bulk TMDs can exist not only in the hexagonal 2H phase but also in trigonal 1T and rhombohedral 3R polymorphs [38]. Moreover, recent investigations showed that transition-metal chalcogenides can form various monolayered structures [39,40,41,42,43,44].

Another interesting class of compounds that are promising materials for lithium-ion batteries are metal–organic frameworks (MOFs) due to their tunable porosity, high surface area, and abundant cavities reducing the strain during charging and discharging processes, resulting in increased service life [45,46,47], The direct utilization of MOFs as anodes in SIBs is hindered by poor electrical conductivity and instability in organic electrolytes [46], but some MOF derivatives are deprived of such shortcomings [46,48]. Despite all the advantages of MOFs, their application is still limited due to the ambiguous principles of their design and insufficient knowledge of the material.

The ability of TMDs to act as an anode in both LIBs and SIBs can be improved by creating a porous structure inspired by MOFs. In the present work, the membrane-like structure of the A’-MoS_2_ monolayer was theoretically examined in terms of application as an anode material for LIBs and NIBs.

## 2. Methods

In this work, all quantum-chemical calculations were based on density-functional theory (DFT) [49,50] and performed within the VASP [51,52,53,54,55] program package. The projected augmented wave (PAW) [56] method was used to describe the interactions between electrons and ion cores. The exchange-correlation functional was calculated via generalized gradient approximation (GGA) in Perdew–Burke–Ernzerhof (PBE) parameterization [57]. The energy cutoff of plane waves was set to be equal to 400 eV. To investigate the dependence of the alkali metal (lithium and sodium) absorption and diffusion on the MoS_2_ surface on the concentration we considered a unit cell of A’-MoS_2_ consisting of 12 sulfur and 6 molybdenum atoms. The first Brillouin zone was sampled according to the Monkhorst–Pack scheme [58], and the k-point mesh of 5 × 3 × 1 was chosen. The relaxation of the atomic geometry was carried out until the maximum values of the energy difference between two electronic steps became less than 10^−6^ eV and between two ionic steps, 10^−3^ eV. The Grimme corrections (DFT-D3) [59] were applied to take into account van der Waals interaction. To avoid the interactions between the periodic images we used a vacuum region of at least 15 Å in non-periodic directions. We used the elastic-band method (NEB) [60,61] combined with DFT calculations to investigate the minimal in energy diffusion pathways. To estimate the dynamic stability of A’-MoS_2_ monolayers we studied the vibrational properties via phonon dispersion calculations based on the density functional perturbation theory. The force-constant matrix was calculated for the 2 × 2 × 1 supercell in the PHONOPY software package [62]. To take into account the crystal symmetry and get rid of the “overdetermination” of the system, the resulting matrix was processed using machine learning algorithms implemented in the Hiphive software package [63]. The VESTA [64] software was used for atomic structure visualization.

## 3. Results

**Figure 1:**
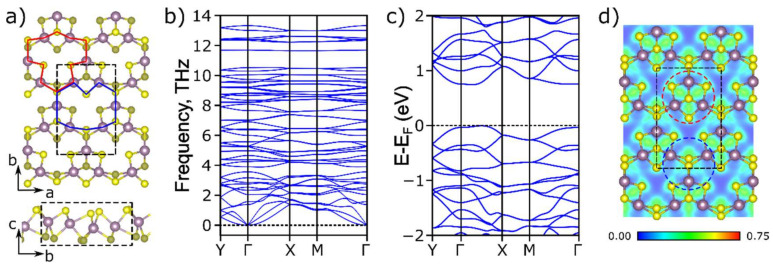
(**a**) Atomic structure, (**b**) phonon dispersion spectra, (**c**) electronic band structure, and (**d**) electron localization function for the total electron density along the *ab* plane for A’-MoS_2_. The unit cell is indicated by black dashed lines. Mo and S atoms are depicted in purple and yellow colors, respectively. Two types of pores in the A’-MoS_2_ structure are marked by red and blue areas in (**a**).

In our work, we considered the MoS_2_ monolayer in the A’ phase, the structure of which was firstly proposed by Gavryushkin et al. [39] for Janus TMDs. The A’-MoS_2_ monolayer is characterized by rhombic symmetry and belongs to the Pmn_2_1 space group with the unit cell vectors of *a* = 6.42 Å and *b* = 9.79 Å. The feature of the A’-MoS_2_ structure is the presence of ordered pores resulting in a lower area density of 2.54·10^−7^ g·cm^−2^ compared with the values for known H (3.03·10^−7^ g·cm^−2^) and T (3.04·10^−7^ g·cm^−2^) phases. The unit cell contains two pores that are equivalent but mirrored concerning the *ab* plane (see Figure 1a red and blue) which leads to the increase of the surface area and facilitates the possibility of the alkali-metal atoms’ diffusion in the perpendicular direction to the layer since the covalent radius of Li and Na atoms is less than the pore diameter. This feature provides more opportunities for the intercalation of alkali-metal atoms and makes the considered structure promising for use as an anode material in metal-ion batteries.

The dynamic stability of the A’-MoS_2_ was investigated by phonon dispersion spectra calculation (see Figure 1b), and the absence of imaginary frequencies revealed the stability of the considered structure. To study the electronic properties of the A’-MoS_2_ we calculated the density of electronic states and the electronic band structure along the path of Y(0, ^1^/_2_, 0) → Γ(0, 0, 0) → X(^1^/_2_, 0, 0) → M(^1^/_2_, ^1^/_2_, 0) → Γ(0, 0, 0) (see Figure 1c). The monolayer was shown to exhibit semiconducting electronic properties with an indirect band gap of 0.74 eV. In comparison, H-MoS_2_ is a semiconductor with a band gap of 1.71 eV, while the T phase is a metal [65]. The *p*-states of the sulfur atom and the molybdenum *d*-states made the main contribution to the valence bond minimum, while the conduction band minima were mainly connected with Mo *d*-states (see Supplementary Figure S1). The semiconducting nature of the A’-MoS_2_ can be attributed to two types of Mo–Mo interactions presented in the structure and associated with different bond lengths. The electron localization function (ELF) [66] displayed a non-zero value on the Mo–Mo bond with the length of 2.73 Å (see Figure 1d, red circle) which indicates the existence of an interaction between these molybdenum atoms also observed in the metallic T phase [67]. On the contrary, in the case of Mo atoms with a bond length of 3.18 and 3.64 Å (see Figure 1d, blue circle) the interaction between Mo atoms was significantly less and the ELF pattern was close to the case of the H phase [68]. Based on the ELF analysis one can conclude that the semiconducting nature of the A’-MoS_2_ arises from the weak Mo–Mo interaction between isolated Mo_3_S_4_ clusters connected through sulfur bridges. The small bandgap of the A’ structure compared to the H phase makes it more convenient for metal-ion batteries due to low barrier electron transfer.

**Figure 2:**
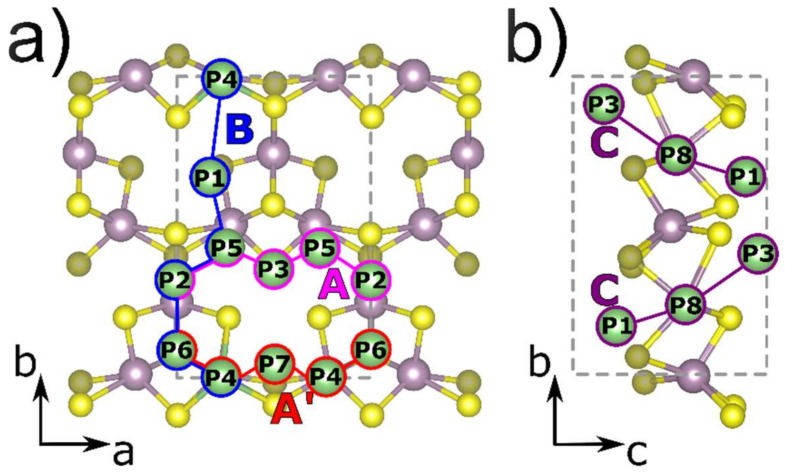
The considered positions of alkali-metal atom (Li, Na) absorption on the surface of the A’-MoS_2_ monolayer and the diffusion pathways: (**a**) A (pink) and B (blue), (**b**) C (red) and D (violet). The unit cell is indicated by the grey dashed lines. Mo, S, and alkali-metal atoms (Li, Na) are depicted in purple, yellow, and green colors, respectively.

To investigate the alkali metal diffusion on the surface of the monolayer the intermediate positions of single alkali-metal atoms corresponding to a local energy minimum on the A’-MoS_2_ structure were evaluated. We considered the positions above the molybdenum atoms (positions P2, P4, and P5), above the sulfur atoms (positions P1, P3, P6, and P7), and in the center of the pore (P8) as indicated in Figure 2a,b.

We calculated the corresponding adsorption energies of a single Li/Na atom ($E_{ads}$) as:

(1) $$E_{ads}=E_{MoS_{2}+1AM}-E_{MoS_{2}}-E_{AM},$$

where $E_{MoS_{2}}$ and $E_{MoS_{2}+1Me}$ are the total energies of pristine 1A’-MoS_2_ and 1A’-MoS_2_ with adsorbed alkali-metal atom, and $E_{AM}$ is the chemical potential of an alkali-metal (AM) atom (Li/Na) calculated from the corresponding BCC crystal. The obtained values of a single alkali-metal atom are presented in Table 1. The negative value of the adsorption energy indicates that it is more energetically favorable for alkali-metal atoms to be adsorbed onto the MoS_2_ surface than to form a crystal.

The most energetically favorable position for both Li and Na atom adsorption corresponds to the position P1 above the three sulfur atoms forming the pore. The most unfavorable site for the Li atom is P7 above a sulfur triangle near the pore. The position P8 in the center of the pore and P6 above the central sulfur in the Mo_3_S_3_ cluster is the second most favorable for lithium atoms while for sodium atoms the position in the center of the pore is the most energetically unfavorable due to steric factors. In all considered cases the adsorption energy was negative meaning that the process of lithium and sodium storage on the surface of A’-MoS_2_ was exothermic. The whole set of calculated positions corresponds to local minima of energy through which the diffusion of alkali-metal atoms can be considered.

All possible pathways of alkali-atom diffusion via corresponding positions on the A’-MoS_2_ surface were considered: two paths along *a* direction of the structure P2→P5→P3→P5→P2 (denoted as A-path, see Figure 2a, pink) and P6→P4→P7→P4′→P6′ (denoted as A’-path, see Figure 2a, blue), the path along *b* direction of the monolayer P4→P1→P5→P2→P6→P4 (denoted as B-path, see Figure 2a, red), as well as perpendicular to the slab surface direction via pore P1→P8→P3 (denoted as C-path, see Figure 2b, violet). Considered diffusion energy profiles are presented in Fig S2. The A-path was the most energetically favorable with the maximum barrier value of 0.16 eV for Li and 0.13 eV for Na atoms between P5 and P2 local minima (see Table 2). The A’-path was limited by the transition from P4 to P7 local minima (0.21 eV for Li and 0.14 for Na) and the P7 was the least energetically favorable among considered positions on the surface of A’-MoS_2_ for both Li and Na atoms (see Table 2). The B-path was limited by transitions from P1 local minimum, which was the most energetically favorable position, by barriers from P1 to P4 (0.46 eV for Li and 0.50 eV for Na) in one direction and from P1 to P5 (0.40 eV for Li and 0.35 for Na) in the reverse direction (see Table 2). Considering the obtained values of diffusion barriers one can conclude that the diffusion of alkali-metal atoms on the surface of A’-MoS_2_ has an anisotropic character and is more likely to occur in the A and A’ directions than in the B direction. In the case of Li-atom diffusion on the surface of the A’-MoS_2_, the obtained values of diffusion barriers were lower than for H-MoS_2_ (0.57 eV [69]), while in the case of Na atom the values for A’-MoS_2_ slightly exceeded the corresponding value for H-MoS_2_ (0.28 eV [69]). In contrast to H-MoS_2,_ the structure of A’-MoS_2_ contains pores, therefore, there is an additional diffusion direction perpendicular to the surface (C-path) for this structure. The diffusion through the pore following the C-path is associated with a local minimum P8, which, in the case of sodium, differed significantly from the most energetically favorable position P1 at 0.71 eV. In addition, to advance the Na atom into the pore relatively high energy barriers must be overcome (1.05 eV from P3 to P8 and 0.82 eV from P1 to P8) because of the steric factor correlated with a bigger ionic radius of the sodium atom compared to the lithium atom. In the case of the lithium atom diffusion through the P8 position, the diffusion barriers were less than through the P1 position along the B-path (see Table 2) which allows us to conclude that Li atoms can exhibit mobility not only on the surface of the A’-MoS_2_ but also can overcome jumps between the slab surfaces.

**Figure 3:**
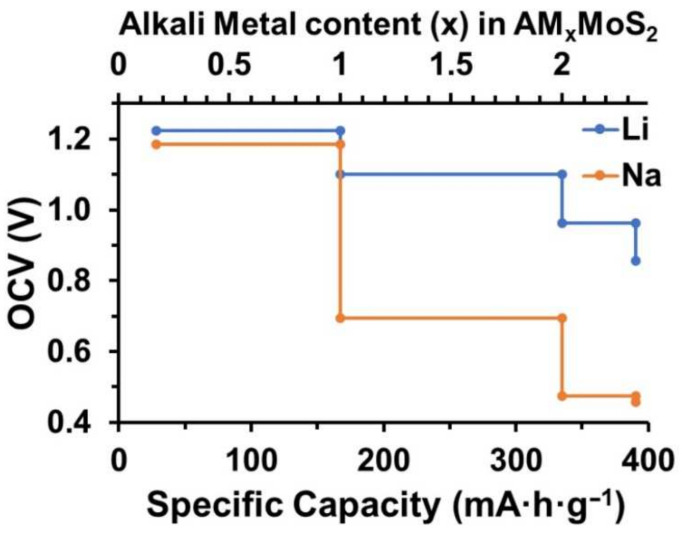
The open circuit voltage profile for A’-MoS_2_ as a function of the theoretical capacity of Li (blue) and Na (orange).

To estimate the electrochemical performance of A’-MoS_2_ anodes we calculated the open circuit voltage (*OCV*) and the value of the theoretical capacity (C). The *OCV* was calculated as:

(2) $$OCV=-\frac{\Delta E}{nze}=-\frac{\;E_{A^{\prime }}\left[MoS_{2}+nMe\right]-E_{A^{\prime }}\left[MoS_{2}\right]-nE\left[Me\right]}{nze},$$

where $E_{A^{\prime }}\left[MoS_{2}+nMe\right]$ and $E_{A^{\prime }}\left[MoS_{2}\right]$ are the total energies of A’-MoS_2_ before and after n alkali-metal atoms adsorption, z is the electronic charge of metal ions (in the case of Li an Na atoms z = 1), and e represent electron charge, while the value of theoretical capacity (C) in mA·h·g^−1^ was calculated using the formula:

(3) $$C=10^{3}\frac{znF}{M_{A^{\prime }}},$$

where F is the Faraday constant (F = 26.801 A·h·mol^−1^) and $M_{A^{\prime }}$ is the mole weight of the A’-MoS_2_ unit cell. The *OCV* profile as a function of theoretical capacity (C) for Li and Na atoms is shown in Figure 3.

The *OCV* for the A’-MoS_2_ monolayer for both Li and Na was higher in comparison to the pristine H-MoS_2_ monolayer [69] at the same range of capacities due to higher absorption energy of alkali-metal atoms and was close to the values for defective H-MoS_2_ containing Mo vacancies [69]. The highest value of A’-MoS_2_ specific capacity denoted to the unit cell (Mo_6_S_12_) with 14 alkali-metal atoms was equal to 390.65 mA·h·g^−1^ for both Li and Na which exceeds the value for the pristine H-MoS_2_ monolayer [32,33,70]. The same value of maximum specific capacity for Na and for Li provided by the A’-MoS_2_ area density makes the A’-MoS_2_ membrane promising not only for LIBs and but also for SIBs without capacity loss.

## 4. Conclusions

To summarize, the novel porous A’-MoS_2_ monolayer was investigated via DFT calculations. The membrane-like structure of A’-MoS_2_ made possible Li and Na diffusion in all directions, but Na diffusion through the pore was limited by the high value of the diffusion barrier (1.05 eV) due to the steric factor. At the same time, the barrier for Li atom (0.38 eV) was lower than the highest one for diffusion on the surface (0.46 eV). The *OCV* values of the novel A’-MoS_2_ monolayer were found to be in the same range as H-MoS_2_ with Mo vacancies. The theoretical specific capacity was ~391 mA·h·g^−1^, which was 17% higher than the maximum filling of 1T and 2H phases (~334 mA·h·g^−1^) by Li atoms. Due to area density, the Na atom can fill the A’-MoS_2_ monolayer with a same specific capacity which characterized the A’ phase as a good candidate for the anode in LIBs/SIBs.

## Figures and Tables

**Table 1:** The adsorption energy of a single alkali-metal atom on the A’-MoS_2_ surface and the energy difference calculated relative to position P1.

Position	$E_{ads}\;(\mathrm{eV})$		Relative Energy (eV)	
	Li	Na	Li	Na
P1	−1.22	−1.18	0.00	0.00
P2	−1.00	−0.87	0.25	0.35
P3	−1.01	−0.93	0.21	0.25
P4	−0.91	−0.80	0.31	0.39
P5	−0.99	−0.93	0.24	0.26
P6	−1.04	−0.86	0.19	0.34
P7	−0.79	−0.16	0.43	0.48
P8	−1.03	−0.43	0.19	0.71

**Table 2:** The energy-barrier values for the diffusion of alkali-metal atom (Li or Na) on the surface of the A’-MoS_2_ monolayer and through the pore.

Transition Path			Li		Na	
			Direct	Reverse	Direct	Reverse
On the surface	A	P2→P5	0.16	0.16	0.06	0.13
		P5→P3	0.11	0.13	0.06	0.05
	A’	P6→P4	0.19	0.07	0.08	0.02
		P4→P7	0.21	0.09	0.14	0.04
	B	P4→P1	0.14	0.46	0.11	0.50
		P1→P5	0.40	0.17	0.35	0.10
		P5→P2	0.16	0.16	0.13	0.06
		P2→P6	0.21	0.26	0.16	0.15
		P6→P4	0.19	0.07	0.08	0.02
Through the slab	C	P3→P8	0.22	0.38	1.05	0.55
		P8→P1	0.22	0.28	0.07	0.82

## Data Availability

Not applicable.

## Supplementary Materials

The following supporting information can be downloaded at: https://www.mdpi.com/article/10.3390/membranes12111156/s1, Figure S1: Density of electronic state structures resolved by d-orbitals for Mo atoms and by p-orbitals for S atoms for A’-MoS2; Figure S2: Diffusion energy profiles for Li (blue) and Na (orange) atoms’ diffusion barriers in different considered directions for A’-MoS_2_.

## References

[1] Liang Y., Dong H., Aurbach D., Yao Y. (2020). Current Status and Future Directions of Multivalent Metal-Ion Batteries. Nat. Energy.

[2] Shea J.J., Luo C. (2020). Organic Electrode Materials for Metal Ion Batteries. ACS Appl. Mater. Interfaces.

[3] Nitta N., Wu F., Lee J.T., Yushin G. (2015). Li-Ion Battery Materials: Present and Future. Mater. Today.

[4] Lee H.-M., Ghovanloo M., Bhunia S., Majerus S., Sawan M. (2015). Energy management integrated circuits for wireless power transmission. Implantable Biomedical Microsystems.

[5] Tarascon J.-M. (2010). Key Challenges in Future Li-Battery Research. Philos. Trans. R. Soc. A Math. Phys. Eng. Sci..

[6] Nayak P.K., Yang L., Brehm W., Adelhelm P. (2018). From Lithium-Ion to Sodium-Ion Batteries: Advantages, Challenges, and Surprises. Angew. Chem. Int. Ed..

[7] Slater M.D., Kim D., Lee E., Johnson C.S. (2013). Sodium-Ion Batteries. Adv. Funct. Mater..

[8] Ellis B.L., Nazar L.F. (2012). Sodium and Sodium-Ion Energy Storage Batteries. Curr. Opin. Solid State Mater. Sci..

[9] Eftekhari A., Kim D.-W. (2018). Sodium-Ion Batteries: New Opportunities beyond Energy Storage by Lithium. J. Power Sources.

[10] Peng L., Zhu Y., Chen D., Ruoff R.S., Yu G. (2016). Two-Dimensional Materials for Beyond-Lithium-Ion Batteries. Adv. Energy Mater..

[11] Yang Y., Liu X., Zhu Z., Zhong Y., Bando Y., Golberg D., Yao J., Wang X. (2018). The Role of Geometric Sites in 2D Materials for Energy Storage. Joule.

[12] Tang X., Ye H., Liu W., Liu Y., Guo Z., Wang M. (2021). Lattice-Distorted Lithiation Behavior of a Square Phase Janus MoSSe Monolayer for Electrode Applications. Nanoscale Adv..

[13] Bahari Y., Mortazavi B., Rajabpour A., Zhuang X., Rabczuk T. (2021). Application of Two-Dimensional Materials as Anodes for Rechargeable Metal-Ion Batteries: A Comprehensive Perspective from Density Functional Theory Simulations. Energy Storage Mater..

[14] Zhang H., Yang Y., Ren D., Wang L., He X. (2021). Graphite as Anode Materials: Fundamental Mechanism, Recent Progress and Advances. Energy Storage Mater..

[15] Tarascon J.-M., Armand M. (2001). Issues and Challenges Facing Rechargeable Lithium Batteries. Nature.

[16] Zhang J., Cao H., Tang X., Fan W., Peng G., Qu M. (2013). Graphite/Graphene Oxide Composite as High Capacity and Binder-Free Anode Material for Lithium Ion Batteries. J. Power Sources.

[17] Senyshyn A., Mühlbauer M.J., Dolotko O., Ehrenberg H. (2015). Low-Temperature Performance of Li-Ion Batteries: The Behavior of Lithiated Graphite. J. Power Sources.

[18] Wen Y., He K., Zhu Y., Han F., Xu Y., Matsuda I., Ishii Y., Cumings J., Wang C. (2014). Expanded Graphite as Superior Anode for Sodium-Ion Batteries. Nat. Commun..

[19] Stevens D.A., Dahn J.R. (2001). The Mechanisms of Lithium and Sodium Insertion in Carbon Materials. J. Electrochem. Soc..

[20] Cao Y., Xiao L., Sushko M.L., Wang W., Schwenzer B., Xiao J., Nie Z., Saraf L.V., Yang Z., Liu J. (2012). Sodium Ion Insertion in Hollow Carbon Nanowires for Battery Applications. Nano Lett..

[21] Moriwake H., Kuwabara A., Fisher C.A.J., Ikuhara Y. (2017). Why Is Sodium-Intercalated Graphite Unstable?. RSC Adv..

[22] Liu Y., Merinov B.V., Goddard W.A. (2016). Origin of Low Sodium Capacity in Graphite and Generally Weak Substrate Binding of Na and Mg among Alkali and Alkaline Earth Metals. Proc. Natl. Acad. Sci. USA.

[23] Chepkasov I.V., Ghorbani-Asl M., Popov Z.I., Smet J.H., Krasheninnikov A.V. (2020). Alkali Metals inside Bi-Layer Graphene and MoS2: Insights from First-Principles Calculations. Nano Energy.

[24] Jian Z., Luo W., Ji X. (2015). Carbon Electrodes for K-Ion Batteries. J. Am. Chem. Soc..

[25] Tapia-Ruiz N., Armstrong A.R., Alptekin H., Amores M.A., Au H., Barker J., Boston R., Brant W.R., Brittain J.M., Chen Y. (2021). 2021 Roadmap for Sodium-Ion Batteries. J. Phys. Energy.

[26] Xu X., Li F., Zhang D., Liu Z., Zuo S., Zeng Z., Liu J. (2022). Self-Sacrifice Template Construction of Uniform Yolk–Shell ZnS@C for Superior Alkali-Ion Storage. Adv. Sci..

[27] Yuan J., Mu M., Xu X., Gan Y., He H., Zhang X., Li X., Kuang F., Li H., Liu J. (2022). Three-Dimensional Porous FeS@ N Doped Carbon Nanosheets for High-Rate and High-Stable Sodium/Potassium Storage. Compos. Part B Eng..

[28] Chen B., Chao D., Liu E., Jaroniec M., Zhao N., Qiao S.-Z. (2020). Transition Metal Dichalcogenides for Alkali Metal Ion Batteries: Engineering Strategies at the Atomic Level. Energy Environ. Sci..

[29] Yun Q., Li L., Hu Z., Lu Q., Chen B., Zhang H. (2020). Layered Transition Metal Dichalcogenide-Based Nanomaterials for Electrochemical Energy Storage. Adv. Mater..

[30] He H., Lu P., Wu L., Zhang C., Song Y., Guan P., Wang S. (2016). Structural Properties and Phase Transition of Na Adsorption on Monolayer MoS2. Nanoscale Res. Lett..

[31] Wang L., Shih E.-M., Ghiotto A., Xian L., Rhodes D.A., Tan C., Claassen M., Kennes D.M., Bai Y., Kim B. (2020). Correlated Electronic Phases in Twisted Bilayer Transition Metal Dichalcogenides. Nat. Mater..

[32] David L., Bhandavat R., Barrera U., Singh G. (2015). Polymer-Derived Ceramic Functionalized MoS_2_ Composite Paper as a Stable Lithium-Ion Battery Electrode. Sci. Rep..

[33] Liu T., Jin Z., Liu D.-X., Du C., Wang L., Lin H., Li Y. (2020). A Density Functional Theory Study of High-Performance Pre-Lithiated MS_2_ (M = Mo, W, V) Monolayers as the Anode Material of Lithium Ion Batteries. Sci. Rep..

[34] Mikhaleva N.S., Visotin M.A., Kuzubov A.A., Popov Z.I. (2017). VS2/Graphene Heterostructures as Promising Anode Material for Li-Ion Batteries. J. Phys. Chem. C.

[35] Yang F., Feng X., Glans P.-A., Guo J. (2021). MoS_2_ for beyond Lithium-Ion Batteries. APL Mater..

[36] Zhang X., Shi H., Liu L., Min C., Liang S., Xu Z., Xue Y., Hong C., Cai Z. (2022). Construction of MoS_2_/Mxene Heterostructure on Stress-Modulated Kapok Fiber for High-Rate Sodium-Ion Batteries. J. Colloid Interface Sci..

[37] Kulka A., Hanc A., Walczak K., Płotek J., Sun J., Lu L., Borca C., Huthwelker T. (2022). Direct Evidence of an Unanticipated Crystalline Phase Responsible for the High Performance of Few-Layered-MoS_2_ Anodes for Na-Ion Batteries. Energy Storage Mater..

[38] Barik G., Pal S. (2020). 2D Square Octagonal Molybdenum Disulfide: An Effective Anode Material for LIB/SIB Applications. Adv. Theory Simul..

[39] Gavryushkin P., Sagatov N., Sukhanova E., Medrish I., Popov Z. (2022). Janus Structures of SMoSe and SVSe Compositions with Low Enthalpy and Unusual Crystal Chemistry. J. Appl. Cryst..

[40] Zhang J., Xia Y., Wang B., Jin Y., Tian H., Ho W.k., Xu H., Jin C., Xie M. (2020). Single-Layer Mo_5_Te_8_—A New Polymorph of Layered Transition-Metal Chalcogenide. 2D Mater..

[41] Wang X., Guan X., Ren X., Liu T., Huang W., Cao J., Jin C. (2020). Deriving 2D M2X3 (M = Mo, W, X = S, Se) by Periodic Assembly of Chalcogen Vacancy Lines in Their MX2 Counterparts. Nanoscale.

[42] Chepkasov I.V., Sukhanova E.V., Kvashnin A.G., Zakaryan H.A., Aghamalyan M.A., Mamasakhlisov Y.S., Manakhov A.M., Popov Z.I., Kvashnin D.G. (2022). Computational Design of Gas Sensors Based on V_3_S_4_ Monolayer. Nanomaterials.

[43] Sukhanova E.V., Kvashnin A.G., Agamalyan M.A., Zakaryan H.A., Popov Z.I. (2022). Map of Two-Dimensional Tungsten Chalcogenide Compounds (W–S, W–Se, W–Te) Based on USPEX Evolutionary Search. JETP Lett..

[44] Sukhanova E., Kvashnin A., Bereznikova L., Zakaryan H., Aghamalyan M., Kvashnin D., Popov Z. (2022). 2D-Mo_3_S_4_ Phase as Promising Contact for MoS_2_. Appl. Surf. Sci..

[45] Kung C.-W., Han P.-C., Chuang C.-H., Wu K.C.-W. (2019). Electronically Conductive Metal–Organic Framework-Based Materials. APL Mater..

[46] Zou G., Hou H., Ge P., Huang Z., Zhao G., Yin D., Ji X. (2018). Metal-Organic Framework-Derived Materials for Sodium Energy Storage. Small.

[47] Dang S., Zhu Q.-L., Xu Q. (2018). Nanomaterials Derived from Metal–Organic Frameworks. Nat. Rev. Mater..

[48] Li X., He C., Zheng J., Ye W., Yin W., Tang B., Rui Y. (2020). Preparation of Promising Anode Materials with Sn-MOF as Precursors for Superior Lithium and Sodium Storage. J. Alloy. Compd..

[49] Hohenberg P., Kohn W. (1964). Inhomogeneous Electron Gas. Phys. Rev..

[50] Kohn W., Sham L.J. (1965). Self-Consistent Equations Including Exchange and Correlation Effects. Phys. Rev..

[51] Hafner J. (2008). Ab-Initio Simulations of Materials Using VASP: Density-Functional Theory and Beyond. J. Comput. Chem..

[52] Kresse G., Furthmüller J., Hafner J. (1994). Theory of the Crystal Structures of Selenium and Tellurium: The Effect of Generalized-Gradient Corrections to the Local-Density Approximation. Phys. Rev. B.

[53] Kresse G., Furthmüller J. (1996). Efficiency of Ab-Initio Total Energy Calculations for Metals and Semiconductors Using a Plane-Wave Basis Set. Comput. Mater. Sci..

[54] Kresse G., Furthmüller J. (1996). Efficient Iterative Schemes for Ab Initio Total-Energy Calculations Using a Plane-Wave Basis Set. Phys. Rev. B.

[55] Kresse G., Joubert D. (1999). From Ultrasoft Pseudopotentials to the Projector Augmented-Wave Method. Phys. Rev. B.

[56] Blöchl P.E. (1994). Projector Augmented-Wave Method. Phys. Rev. B.

[57] Perdew J.P., Burke K., Ernzerhof M. (1996). Generalized Gradient Approximation Made Simple. Phys. Rev. Lett..

[58] Monkhorst H.J., Pack J.D. (1976). Special Points for Brillouin-Zone Integrations. Phys. Rev. B.

[59] Grimme S., Antony J., Ehrlich S., Krieg H. (2010). A Consistent and Accurate Ab Initio Parametrization of Density Functional Dispersion Correction (DFT-D) for the 94 Elements H-Pu. J. Chem. Phys..

[60] Henkelman G., Jónsson H. (2000). Improved Tangent Estimate in the Nudged Elastic Band Method for Finding Minimum Energy Paths and Saddle Points. J. Chem. Phys..

[61] Henkelman G., Uberuaga B.P., Jónsson H. (2000). A Climbing Image Nudged Elastic Band Method for Finding Saddle Points and Minimum Energy Paths. J. Chem. Phys..

[62] Togo A., Tanaka I. (2015). First Principles Phonon Calculations in Materials Science. Scr. Mater..

[63] Eriksson F., Fransson E., Erhart P. (2019). The Hiphive Package for the Extraction of High-Order Force Constants by Machine Learning. Adv. Theory Simul..

[64] Momma K., Izumi F. (2011). VESTA 3 for Three-Dimensional Visualization of Crystal, Volumetric and Morphology Data. J. Appl. Cryst..

[65] Kumar A., Ahluwalia P.K. (2012). Electronic Structure of Transition Metal Dichalcogenides Monolayers 1H-MX_2_ (M = Mo, W; X = S, Se, Te) from Ab-Initio Theory: New Direct Band Gap Semiconductors. Eur. Phys. J. B.

[66] Savin A., Nesper R., Wengert S., Fässler T.F. (1997). ELF: The Electron Localization Function. Angew. Chem. Int. Ed..

[67] Lin G., Ju Q., Guo X., Zhao W., Adimi S., Ye J., Bi Q., Wang J., Yang M., Huang F. (2021). Intrinsic Electron Localization of Metastable MoS2 Boosts Electrocatalytic Nitrogen Reduction to Ammonia. Adv. Mater..

[68] Li Y., Li Y.-L., Araujo C.M., Luo W., Ahuja R. (2013). Single-Layer MoS_2_ as an Efficient Photocatalyst. Catal. Sci. Technol..

[69] Barik G., Pal S. (2019). Defect Induced Performance Enhancement of Monolayer MoS_2_ for Li- and Na-Ion Batteries. J. Phys. Chem. C.

[70] Su J., Pei Y., Yang Z., Wang X. (2014). Ab Initio Study of Graphene-like Monolayer Molybdenum Disulfide as a Promising Anode Material for Rechargeable Sodium Ion Batteries. RSC Adv..

