# Supplementary material for: A Novel Membrane-like 2D A’-MoS2 as Anode for Lithium- and Sodium-Ion Batteries
Source: Membranes (Basel). 2022 Nov 16;12(11):1156. doi: 10.3390/membranes12111156 (PMC9693981; doi:10.3390/membranes12111156)
Supplement: Supplementary file 1 [file membranes-12-01156-s001.zip › membranes-2014185-supplementary.pdf]

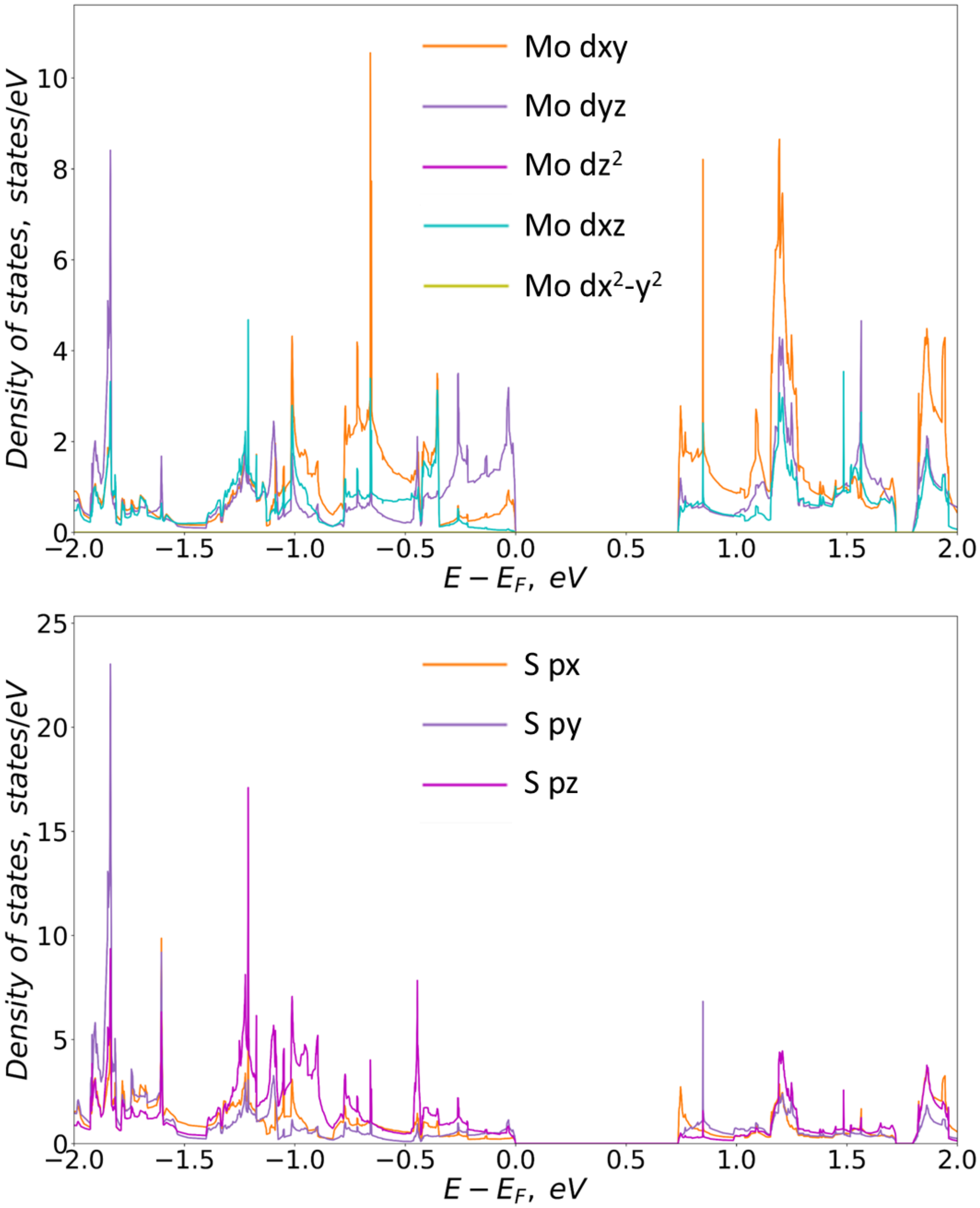

Figure S1. Density of electronic states structure resolved by d-orbitals for Mo atoms and by p-orbitals for S atoms for A'-MoS_2.

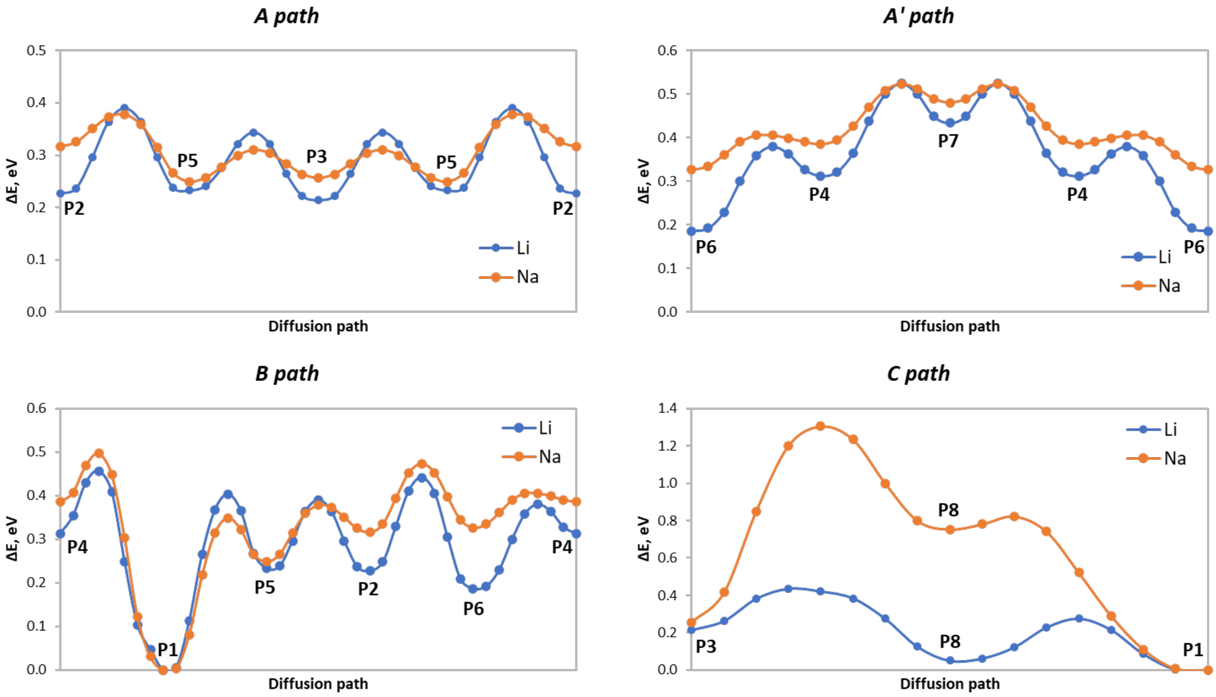

Figure S2. Diffusion energy profiles for Li (blue) and Na (orange) atoms diffusion barriers in different considered directions for A'-MoS_2.
